# Supplementary material for: Interactions between Pseudomonas aeruginosa and six opportunistic pathogens cover a broad spectrum from mutualism to antagonism
Source: Environ Microbiol Rep. 2024 Oct 2;16(5):e70015. doi: 10.1111/1758-2229.70015 (PMC11445780; doi:10.1111/1758-2229.70015)
Supplement: Supplementary file 3 — Data S3. Supporting information. [file EMI4-16-e70015-s006.docx]

**Supporting Information**

**Interactions between *Pseudomonas aeruginosa* and six opportunistic pathogens cover a broad spectrum from mutualism to antagonism**

Clémentine Laffont^1^, Tobias Wechsler^1^, Rolf Kümmerli^1^

^1^ Department of Quantitative Biomedicine, University of Zurich, Winterthurerstrasse 190, 8057 Zürich, Switzerland

Corresponding authors:

[clementine.laffont@gmail.com](mailto:clementine.laffont@gmail.com)

[rolf.kuemmerli@uzh.ch](mailto:rolf.kuemmerli@uzh.ch)

Supporting Information contains:

- 4 supplementary figures: Figure S1-S4 (in this file)
- 8 supplementary tables: Table S1-S8 (in a separate excel file called “Supplementary Tables”)
- 6 supplementary movies: Movie S1-S6 (as individual movies)
- 6 supplementary data: Data S1-S6 (in a separate excel file called “Supplementary Data”)
- 1 supplementary method (including 5 scripts in addition to a separate file called “Supplementary Method”).

**Supplementary figures:**


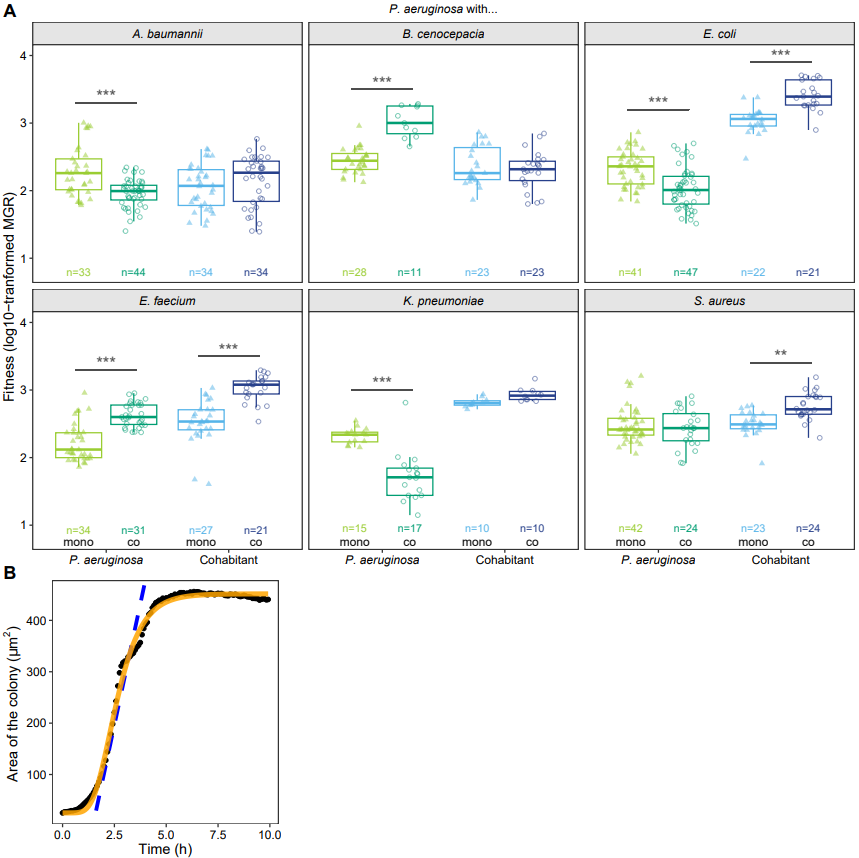


**Figure S1: The maximum growth rate is affected when *P. aeruginosa* is co-cultured with other pathogens. (A)** The boxplots depict the log10-transformed fitness (MGR: maximum growth rate) of *P. aeruginosa* (green) and its cohabitants (blue) in monocultures (light triangles) and co-cultures (dark circles). n-values indicate the total number of colonies tracked for each species combination. Two-way ANOVAs were used in combination with TukeyHSD *post-hoc* tests to examine fitness differences between mono- and co-cultures for *P. aeruginosa* and its cohabitants. Asterisks show the level of significance: * p_adj_ < 0.05, ** p_adj_ < 0.01, *** p_adj_ < 0.001. (B) Gompertz models were used to quantify the maximum growth rate from the growth curves. The black points depict the real data, while the orange curve represents the model fit. The blue dashed line shows the maximum slope of the curve (based on the fitted model) used to calculate the maximum growth rate.


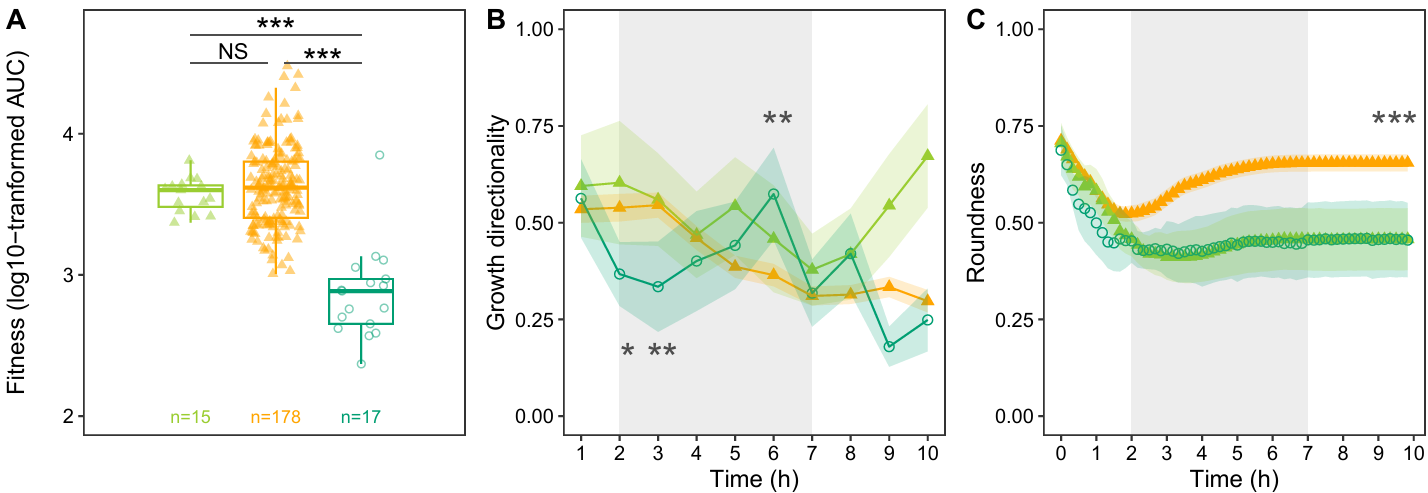


**Figure S2: The roundness of *P. aeruginosa* monocultures colonies is affected by *K. pneumoniae* colonies growing on nearby, yet physically separated, agarose pads.** (A) Fitness, (B) growth directionality and (C) roundness of *P. aeruginosa* colonies grown (i) as monocultures in the absence of *K. pneumoniae* (orange triangles) within the same gene frame, (ii) as monocultures in the presence of *K. pneumoniae* within the same gene frame (light green triangles), and (iii) as co-cultures with *K. pneumoniae* (dark green circles). Data points and shaded areas (B and C) show the means and the 95% confidence intervals across all colonies experiencing the same conditions. The grey shaded areas show the time window (2^nd^ to 7^th^ hour) during which most of the growth occurred. The boxplots (A) depict the log10-transformed values of the AUC (area under the growth curve). Asterisks show the level of significance from the TukeyHSD *post-hoc* tests: * p < 0.05, ** p < 0.01, *** p < 0.001. NS means non-significant.

The comparisons show that *P. aeruginosa* monoculture fitness is not affected by the absence or presence of *K. pneumoniae* monocultures within the same gene frame (A, light green versus orange triangles). Similarly, there is no difference in the growth directionality (B) during the time of actual growth between the two types of monocultures (i) and (ii) (except one time point: 5h), although there are certain differences (at 2h, 3h and 6h) when comparing the monocultures without *K. pneumoniae* (i) to the co-cultures (iii). Most importantly, we observed a clear difference between the two *P. aeruginosa* monocultures (i) and (ii) regarding colony roundness (C). Specifically, *P. aeruginosa* colony roundness was high in all experiments without *K. pneumoniae*, whereas *P. aeruginosa* colony roundness dropped in all experiments with *K. pneumoniae*, even when *K. pneumoniae* grew on physically separated agarose pads. This finding suggests that *K. pneumoniae* produces volatiles that impact *P. aeruginosa* colonies on adjacent pads.


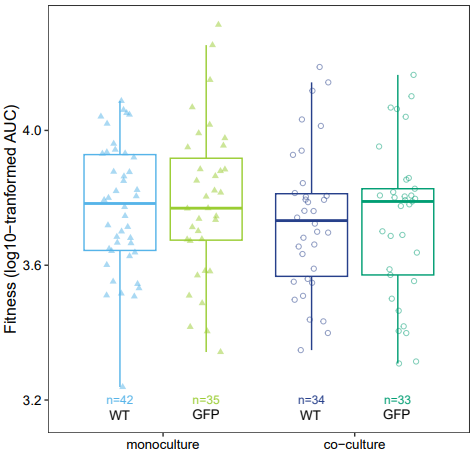


**Figure S3: The growth of the *P. aeruginosa* GFP-tagged strain used in the study is similar to the wild type strain.** The boxplots depict the log10-transformed fitness (AUC: area under the colony growth curve) of *P. aeruginosa* tagged with GFP (green) and the wild type (WT - blue) in monocultures (light triangles) and co-cultures (dark circles). n-values indicate the total number of colonies tracked for each conditions. A two-way ANOVA was used in combination with Tukey HSD *post-hoc* tests to examine fitness differences between mono- and co-cultures for *P. aeruginosa* GFP and WT strains. No significant growth differences between the two strains were observed.


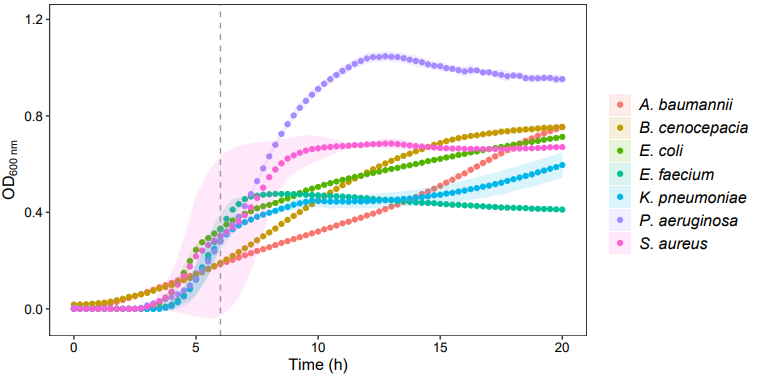


**Figure S4: Cultures of all species were collected at the exponential growth phase to prepare samples for microscopy experiments.** Following growth overnight in TSB, all pathogen species were diluted and re-grown for 6h in fresh medium in order to reach their exponential growth phase. The data represent the means (points) and the 95% confidence intervals (shaded areas) calculated from 3 biological replicates. Growth was tracked in 1.5mL volumes of TSB 70% in 24-well plates, incubated at 37°C in a plate reader (Tecan). Absorbance measurements were taken at 600nm every 15min during 20 hours. Prior to each measurement, cultures were shaken (orbital shaking of 3mm) for 30 seconds.
