# Supplementary material for: Interactions between Pseudomonas aeruginosa and six opportunistic pathogens cover a broad spectrum from mutualism to antagonism
Source: Environ Microbiol Rep. 2024 Oct 2;16(5):e70015. doi: 10.1111/1758-2229.70015 (PMC11445780; doi:10.1111/1758-2229.70015)
Supplement: Supplementary file 4 — Data S4. Supporting information. [file EMI4-16-e70015-s007.docx]

**Supplementary method**

**Interactions between *Pseudomonas aeruginosa* and six opportunistic pathogens cover a broad spectrum from mutualism to antagonism**

Clémentine Laffont^1^, Tobias Wechsler^1^, Rolf Kümmerli^1^

^1^ Department of Quantitative Biomedicine, University of Zurich, Winterthurerstrasse 190, 8057 Zürich, Switzerland

Corresponding authors:

[clementine.laffont@gmail.com](mailto:clementine.laffont@gmail.com)

[rolf.kuemmerli@uzh.ch](mailto:rolf.kuemmerli@uzh.ch)

- This file contains a step-by-step protocol of our semi-automated colony tracking pipeline. It is associated with 5 scripts (to be used in FIJI):
  - batch_unpack_vsi.py
  - drift_correction.py
  - segment_colonies_batch.py
  - colony_seg_correction.py
  - measure_colonies.py

**Description:**

This workflow can be used to follow microcolonies growing on agarose pads. It segments the microcolonies as a whole, tracks them over time, and provides tools to correct the segmentation and tracking.

**1. Pre-Processing:**

The images can be converted to individual tiff images with the batch_unpack_vsi.py script or by importing the file into FIJI and exporting them as tiff images. The images have to be exported as individual TIFF stacks for each channel, with the phase contrast stack ending in _phase.tif and the fluorescence stack in _fluor.tif respectively. In a first step, we corrected drift between time points.

- Usage:
  - The drift correction is already integrated in the batch_unpack_vsi.py script. But in order for it to work, the drift_correction.py script must be in the same directory as the batch_unpack_vsi.py script.
  - After starting the script in FIJI, select the parent directory containing all your VSI-files.
  - The script creates a new directory with subdirectories for each position.

**2. Threshold Segmentation:**

The next step was to segment individual colonies based on the phase contrast images. The image background was subtracted using the rolling ball algorithm in FIJI (radius = 40 pixels, ca. 4 µm) to correct for uneven illumination and increase the contrast between background and colonies. To smooth over gaps between cells within a colony, we applied a Gaussian filter (sigma = 10 pixels, ca. 1 µm). We used the default automatic threshold function to create a segmentation mask and subsequently regions of interest (ROI). This step can be performed with the segment_colonies_batch.py script.

- Usage:
  - To run the segmentation on all positions, run the segment_colonies_batch.py script in FIJI and select the parent directory containing all subdirectories of the individual positions.
  - A ROI folder gets created within each position directory.
  - The ROIs are stored as zip files for each time point.

**3. Correct Segmentation and Tacking:**

In a next step, we manually corrected our segmentation. The manual correction consisted of separating merging colonies based on fluorescence images (in the case of mixed positions) or by eye (for colonies of the same strain) and combining colonies that merge early in the time-lapse. We further excluded colonies that partially grew out of the field of view and corrected or discarded colonies with inaccurate segmentations. Colonies were tracked over time based on their overlap with the segmentation of the previous time point. We discarded colonies that had no overlap with any colonies from the previous time point. The colony_seg_correction.py script can assist you in performing these steps.

- Usage:

After starting the script in FIJI, select the folder of the position you want to correct. You can add or delete ROIs in the ROI Manager. In addition, you can use the Command launcher to perform the following actions.

- - Extend: extend a ROI with an overlapping manual selection.
  - Clone: copy the currently selected ROI to the next frame.
  - Merge: combine two selected ROI to one.
  - Split: draw a selection over a ROI and split it along the selection.
  - Save: save the corrected ROIs in a folder with a time-stamp.
  - Track: track colonies over time. Note that all colonies that are present in some but not all time points will be removed from all time points.

While correcting the ROIs, they are stored in a new folder (ROI_CHECK) and will be moved to another upon completion of the correction (ROI_DONE). If you want to change the already corrected ROIs further, you can move the zip files from the ROI_DONE folder into the original ROI folder. Note that if the ROIs are not shown in the image, you have to select More >> Options in the ROI Manager and uncheck the box ‘Associate "Show ALL" ROIs with slices’. Here, you can also choose to ‘Use ROI names as labels’ to display the actual ROI names on the image.

**4. Measurements:**

We measured the area (in pixels), the X and Y coordinates of the centroids, the roundness and the fluorescence for all the colonies in each time point. In addition, we also manually measured the area of the double layers at the last time point.

- Usage:
  - Run the measure_colonies.py script in FIJI and select the position directory you want to measure.
  - You can tag a colony as fluorescent by selecting it at any time point and pressing tag in the command launcher. This adds a *p* in front of the ROI name.
  - To measure the double layer in the last time point you can manually add a ROI.
